# Structural Biology of Bacterial Pathogenesis

**DOI:** 10.3201/eid1202.05139

**Published:** 2006-02

**Authors:** Dennis Stevens

**Affiliations:** * Veterans Administration Medical Center, Boise, Idaho, USA

**Keywords:** bacterial pathogenesis, vaccine development, sortases

Research into the pathogenesis of microbial infections has a long and fruitful history, rich with elucidation of mechanisms that have resulted in better treatments and new strategies for vaccine development. Stanley Falkow's investigations into the intimate relationships between bacteria and host cells followed his comment that, "The microbe is just trying to make a living." Structural Biology of Bacterial Pathogenesis, by Gabriel Waksman, Michael Caparon, and Scott Hultgren, is a state-of-the-art treatise describing the known molecular mechanisms by which bacterial pathogens actively probe, sense, and respond to their environment through 2-component systems and through sigma and anti-sigma factors. In addition, the authors describe in great detail the recognition of host receptors by pili and the pilus biogenesis by chaperon-user pathways. The chapter on the role of sortases on surface expression of surface proteins among gram-positive bacteria is comprehensive and well written. Four excellent chapters describe 6 secretion systems among bacterial pathogens and elucidate the specific mechanisms by which bacterial pathogens usurp intracellular mechanisms of the host cell.

This book is enjoyable to read, is extensively referenced, and has 52 superb structural models in full color, based in part on x-ray crystallography. Unfortunately, these plates are all in 1 section and require the reader to page back and forth from specific chapters to the "core color plates." This book is not a compendium of bacterial toxins and virulence factors but rather a selection of molecular mechanisms of host-parasite interaction. This is a unique book that will be a valuable asset for researchers in the field of pathogenesis, graduate students, faculty who teach microbial pathogenesis, biotech companies, and pharmaceutical companies involved in antimicrobial drug or vaccine development.

The sophisticated molecular mechanisms that pathogenic bacteria have developed through their evolution with the human host, as described in this book, are credible evidence that our adversaries, the microbes, are doing better than "just making a living."

